# The elastic theory of shells using geometric algebra

**DOI:** 10.1098/rsos.170065

**Published:** 2017-03-08

**Authors:** A. L. Gregory, J. Lasenby, A. Agarwal

**Affiliations:** Cambridge University Engineering Department, Trumpington Street, Cambridge CB2 1PZ, UK

**Keywords:** shells, elasticity, geometric algebra

## Abstract

We present a novel derivation of the elastic theory of shells. We use the language of geometric algebra, which allows us to express the fundamental laws in component-free form, thus aiding physical interpretation. It also provides the tools to express equations in an arbitrary coordinate system, which enhances their usefulness. The role of moments and angular velocity, and the apparent use by previous authors of an unphysical angular velocity, has been clarified through the use of a bivector representation. In the linearized theory, clarification of previous coordinate conventions which have been the cause of confusion is provided, and the introduction of prior strain into the linearized theory of shells is made possible.

## 1. Introduction

Thin shells have been an active subject of research for some considerable time, however, in attempting to understand the self-excited oscillations of flexible tubes, we have had difficulties finding a complete and rational theory in which the underlying physical principles are clear, and which is easy to apply to the practical problem at hand. Specifically, it was found that in order to have a full understanding of the assumptions of various shell theories, it was necessary to derive our own from first principles. We found that in doing this we were able to produce a theory with improved clarity, brevity and with explicit results for linearization about a deformed state. For reference, our complete nomenclature is given in table 1.

**Table 1.** Nomenclature.

B	second fundamental form on the reference configuration
b	second fundamental form on the spatial configuration
*B*	reference configuration
*b*	body force per unit mass
C	Cauchy–Green tensor
*c*	body moments per unit mass
*C*_*i*_	principal curvatures of the reference configuration
*c*_*i*_	principal curvatures of the spatial configuration
E	Green–Lagrange strain tensor
*E*_3_	normal vector to the reference configuration
*e*_3_	normal vector to the spatial configuration
{*E*_*A*_}	frame for bivectors on the reference configuration
{*e*_*A*_}	frame for bivectors on the spatial configuration
{*E*^*A*^}	reciprocal frame for bivectors on the reference configuration
{*e*^*A*^}	reciprocal frame for bivectors on the spatial configuration
$\dot{E}$	rate of change of strain tensor
{*E*_*i*_}	frame for the tangent space of the reference configuration
{*e*_*i*_}	frame for the tangent space of the spatial configuration
{*E*^*i*^}	reciprocal frame for the tangent space of the reference configuration
{*e*^*i*^}	reciprocal frame for the tangent space of the spatial configuration
*e*	internal energy per unit mass of the shell, defined on the spatial configuration
*E*	internal energy per unit mass of the shell, defined on the reference configuration
F	deformation gradient
G	metric, or first fundamental form, on the reference configuration
g	metric, or first fundamental form, on the spatial configuration
H	change of curvature tensor
$\dot{H}$	rate of change of the change of curvature tensor
*I*	local pseudoscalar on the reference configuration
*i*	local pseudoscalar on the spatial configuration
*I*_3_	pseudoscalar of three-dimensional Euclidean space
l	strain rate tensor
M	first reference couple-stress tensor
m	couple-stress tensor
**M**	modified first reference couple-stress tensor
N	second reference couple-stress tensor
n	symmetric strain rate tensor
**N**	modified second reference couple-stress tensor
S	second Piola–Kirchhoff stress tensor
*S*	spatial configuration
$\tilde{S}$	modified second Piola–Kirchhoff stress tensor
T	first Piola–Kirchhoff stress tensor
*t*	time
*V*	velocity referred to the reference configuration
*v*	velocity referred to the spatial configuration
V	volume form on the spatial configuration
v	volume form on the reference configuration
w	antisymmetric strain rate tensor
*X*	a point in the reference configuration
{*X*^*i*^}	coordinate system over the reference configuration
{*x*^*i*^}	convected coordinate system over the spatial configuration
$\Gamma_{iB}^{A}$	bivector Christoffel coefficients on the reference configuration
$\Gamma_{ib}^{a}$	Christoffel coefficients on the reference configuration
$\gamma_{iB}^{A}$	bivector Christoffel coefficients on the spatial configuration
$\gamma_{ib}^{a}$	Christoffel coefficients on the spatial configuration
λ_*i*_	principal stretches
*ω*	angular velocity
*ϕ*_*t*_	a motion of the reference configuration
*ρ*	area density of shell
*ρ*_0_	time independent area density of shell
*σ*	Cauchy stress tensor
∇	vector derivative
∂	vector derivative intrinsic to a surface

We also have an interest in applying geometric algebra (GA) [1–3] to new areas of the physical sciences. GA provides the tools to formulate physical laws with as little reference to coordinate systems as possible, which helps with the first aim of clarifying the physical meaning of the equations produced, but it also provides simple tools to allow these equations to be represented in arbitrary coordinate systems, which ensures practical use. This article aims to provide, to our knowledge for the first time in this area, an introduction to shell theory using GA. While in this article we restrict the introduction of GA to the use of bivectors to represent torques and angular velocities, we hope that this will pave the way for more radical developments, such as those completed for the theory of rods [4].

There are a large number (at least 10) of linearized shell theories [5]. The derivations of these theories use a wide variety of notations, coordinate systems and conventions, making it very difficult to compare the assumptions made. In addition, none of the theories reviewed by Leissa [5] allow for prior strain of the shell, which we wish to include for our own analysis. More general shell theories have also been produced, with the most extensive probably that by Naghdi [6], which provides the basis for more modern studies such as [7–9], though the theory of Koiter [10] has also been popular with some authors. While rigorous, these theories have limited practical use. They generally require the use of differential geometry [7,11] whose indicial expressions often hide much of the physical meaning of the equations. The general theory presented by Antman [8] is relegated to a final chapter that does not stand alone, meaning that the entire book must be read to use the shell theory. Naghdi [6] discusses in detail the different advantages of developing a shell theory directly from three-dimensional elasticity or by considering two-dimensional surfaces from the start. Antman [8] restricts his development to the former, but we feel that a more concise and lucid theory can be obtained from the latter.

We aim to use GA to develop an accessible, concise, rational shell theory that can be easily linearized to include pre-strain. In doing this, we will provide new developments in the representation of moments and angular velocities with bivectors, and in the representation of bending, which is where most disagreements occur in linearized shell theories.

## 2. Geometry of surfaces

**Figure 1:**
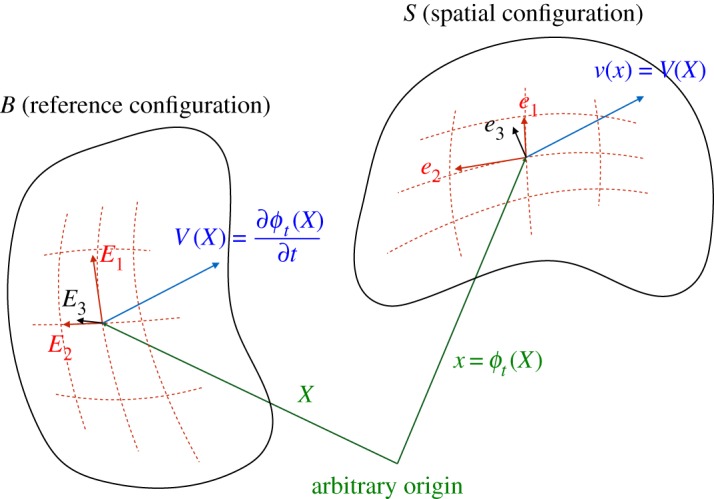
Surface geometry.

Let *B* and *S* be two-dimensional surfaces embedded in three-dimensional Euclidean space $E^{3}$. *B* is the *reference configuration* of the surface, and *S* is the *spatial configuration*, and the two are related by the *motion* *ϕ*_*t*_. At time *t*, the point *X*∈*B* is moved to *ϕ*_*t*_(*X*)∈*S*. Let {*X*^*i*^} be coordinates over *B*, and {*x*^*i*^} be coordinates over *S*. We follow the convention that the indices *i*,*j*,*k*,… run over 1,2, and the indices *a*,*b*,*c*,… run over 1,2,3. We restrict {*x*^*i*^} to be *convected coordinates* such that $x^{i}(x)=X^{i}(\phi_{t}^{-1}(x))$, where *x*∈*S*. We denote the *frame* associated with {*X*^*i*^} by *E*_*i*_=∂*X*/∂*X*^*i*^, and similarly, *e*_*i*_=∂*x*/∂*x*^*i*^. The *reciprocal frames* are denoted by {*E*^*i*^} and {*e*^*i*^}, and are defined to satisfy $E^{i}\cdot E_{j}=e^{i}\cdot e_{j}=\delta_{j}^{i}$. The frames on each configuration are illustrated in figure 1.

The local *pseudoscalars* in the reference and spatial configurations are *I*=*E*_1_∧*E*_2_/|*E*_1_∧*E*_2_| and *i*=*e*_1_∧*e*_2_/|*e*_1_∧*e*_2_|, which satisfy *I*^2^=*i*^2^=−1. We denote the pseudoscalar of $E^{3}$ by *I*_3_. We have defined orientations of both configurations and $E^{3}$ with these pseudoscalars, which allows us to define the *normal vectors* to the surfaces, *E*_3_=−*I*_3_*I* and *e*_3_=−*I*_3_*i*. *E*_3_ and *e*_3_ are unit vectors perpendicular to the other frame vectors, so *E*^3^=*E*_3_ and *e*^3^=*e*_3_. {*E*_*a*_} and {*e*_*a*_} now both form a basis of $E^{3}$. The (scalar) *volume forms* V and v are defined to satisfy $VI=E_{1}\wedge E_{2}$ and $vi=e_{1}\wedge e_{2}$.

The *vector derivative* of $E^{3}$ is denoted by ∇, and the projection of this derivative operator onto either *B* or *S* is denoted by ∂. ∂ can be written locally on *B* as ∂=*E*^*i*^(∂/∂*X*^*i*^), and on *S* as ∂=*e*^*i*^(∂/∂*x*^*i*^). For convenience, we define the notation ∂_*i*_=(∂/∂*X*^*i*^) and ∂_*i*_=(∂/∂*x*^*i*^). It will be clear from context whether differentiation is on the reference or spatial configuration.

Let G(*Y*)=*Y* and g(*y*)=*y* be identity functions, where *Y* is a vector on *B*, and *y* is a vector on *S*. The reason we distinguish these apparently identical linear functions is that they are the *metrics* of the two surfaces, also called the *first fundamental forms*. In component form, we have g_*ab*_=*e*_*a*_⋅g(*e*_*b*_)=*e*_*a*_⋅*e*_*b*_ and g^*ab*^=*e*^*a*^⋅*e*^*b*^. The properties of the reciprocal frame imply that ${g^{a}}_{b}={g_{b}}^{a}=\delta_{b}^{a}$. Analogous results hold for G. The determinant of a function is defined in a coordinate free way by g(i)=(detg)i, from which it is clear that detG=detg=1. However, it is common to define $\det(g_{ij})=g_{11}g_{22}-g_{12}g_{21}$, which is not equal to 1, and in fact encodes important geometric information about the manifold. This is possible because the coordinate free definition of detg corresponds to $\det({g^{i}}_{j})$, and not $\det(g_{ij})$. In fact, we can show that $\det(g_{ij})=v^{2}$. Recalling the definition of v, this demonstrates in a very obvious way that $\sqrt{\det(g_{ij})}$ is a measure of the ‘volume’ spanned by the parallelepiped formed from the basis vectors. GA in this instance provides clarification over the fact that g is simply the identity function, and provides a definition of $v=\sqrt{\det(g_{ij})}$ that makes its geometric significance immediately obvious.

We denote the *second fundamental forms* on *B* and *S* by B and b. b is defined to satisfy $b(y)=-\dot{\partial }(y\cdot {\dot{e}}_{3})$. In component form, we have b_*ij*_=−*e*_*j*_⋅∂*e*_3_/∂*x*^*i*^=*e*_3_⋅∂*e*_*j*_/∂*x*^*i*^, which follows from the fact that *e*_*j*_⋅*e*_3_=0⇒∂_*i*_(*e*_*j*_⋅*e*_3_)=0. From this, it is clear that b_*ij*_=b_*ji*_, and hence b is symmetric, i.e. $b(y)=\bar{b}(y)=-y\cdot \partial e_{3}$. The eigenvalues of b are the *principal curvatures* of the surface, denoted by *c*_1_ and *c*_2_. Analogous results hold for B, whose eigenvalues are denoted by *C*_1_ and *C*_2_. We define the *Christoffel coefficients* $\gamma_{ib}^{a}=e^{a}\cdot \partial e_{b}/\partial x^{i}=-e_{b}\cdot \partial e^{a}/\partial x^{i}$, which follows from the fact that $e^{a}\cdot e_{b}=\delta_{b}^{a}\Rightarrow \partial_{i}(e^{a}\cdot e_{b})=0$. $\gamma_{jk}^{i}$ are the usual coefficients associated with a frame on a manifold. The remaining coefficients are closely related to the second fundamental form by $\gamma_{ij}^{3}=b_{ij}$, $\gamma_{j3}^{i}=-{b^{i}}_{j}$ and $\gamma_{i3}^{3}=0$, as *e*_3_ is a unit vector. We similarly define $\Gamma_{ib}^{a}=E^{a}\cdot \partial E_{b}/\partial X^{i}$.

When considering angular momentum, we will make use of *bivectors*. To do this, we first introduce some notation. The space of all bivectors in $E^{3}$ is spanned by the basis {*e*_(1,3)_=*e*_1_∧*e*_3_,*e*_(2,3)_=*e*_2_∧*e*_3_,*e*_(1,2)_=*e*_1_∧*e*_2_}, and by the reciprocal basis {*e*^(1,3)^=*e*^3^∧*e*^1^,*e*^(2,3)^=*e*^3^∧*e*^2^,*e*^(1,2)^=*e*^2^∧*e*^1^}. We use capital indices to denote bivector indices, and use the convention that the indices *I*,*J*,*K*,… run over (1,3),(2,3), while the indices *A*,*B*,*C*,… run over (1,3),(2,3),(1,2). Hence, the space of bivectors is spanned by {*e*_*A*_} and {*e*^*A*^}. Defined in this way, these basis bivectors satisfy $e^{A}\cdot e_{B}=\delta_{B}^{A}$. In an analogous way to vectors, the general bivector *ω* can be written in component form as *ω*=*ω*_*A*_*e*^*A*^=*ω*^*A*^*e*_*A*_, where *ω*_*A*_=*ω*⋅*e*_*A*_ and *ω*^*A*^=*ω*⋅*e*^*A*^ (here we follow the conventions of [1], eqn. 1-3.18). We can also define the bivector Christoffel coefficients $\gamma_{iB}^{A}=e^{A}\cdot \partial e_{B}/\partial x^{i}$. Given the surface, we have already defined, for which *e*_3_=*e*^3^, these satisfy

2.1 $$\begin{array}{rl} \gamma_{i(1,2)}^{\left(1,2\right)} & =\gamma_{i1}^{1}+\gamma_{i2}^{2},\;\gamma_{i(1,2)}^{\left(1,3\right)}=b_{i2},\;\gamma_{i(1,2)}^{\left(2,3\right)}=-b_{i1},\\ \gamma_{i(1,3)}^{\left(1,2\right)} & =-{b^{2}}_{i},\;\gamma_{i(1,3)}^{\left(1,3\right)}=\gamma_{i1}^{1},\;\gamma_{i(1,3)}^{\left(2,3\right)}=\gamma_{i1}^{2}\\ \;\text{and}\;\gamma_{i(2,3)}^{\left(1,2\right)} & ={b^{1}}_{i},\;\gamma_{i(2,3)}^{\left(1,3\right)}=\gamma_{i2}^{1},\;\gamma_{i(2,3)}^{\left(2,3\right)}=\gamma_{i2}^{2}. \end{array}\}$$

Let m be a bivector valued function of a vector. m(*y*) can be written as m(*y*)=m^*Aa*^*y*_*a*_*e*_*A*_, where *y*_*a*_=*y*⋅*e*_*a*_ and m^*Aa*^=*e*^*A*^⋅m(*e*^*a*^), for example, m^(1,2)2^=(*e*^2^∧*e*^1^)⋅m(*e*^2^).

## 3. Kinematics

We define *X*(*η*) to be a path over *B* parametrized by the scalar *η*. *dX*/*dη* is then a tangent vector to *B*, and we can also obtain a tangent vector to *S*, ∂*ϕ*_*t*_(*X*)/∂*η*. The map between these tangent vectors is denoted by F, and is called the *deformation gradient*. This encodes stretching information for the surface, but also rigid body rotations. Rigid body rotations are not expected to influence constitutive theory, so we construct the *Cauchy–Green tensor* $C(Y)=\bar{F}F(Y)$, which is symmetric. We restrict ourselves to deformations that have an inverse and leave the orientation of *B* unchanged, which means that the eigenvalues of C will be real and positive. It is therefore meaningful to define λ_*i*_ as the square roots of the eigenvalues of C. These are the *principal stretches* of the surface. Using the Cauchy–Green tensor we construct the *Green–Lagrange strain tensor*, $E(Y)=\frac{1}{2}(C(Y)-Y)$, that is only non-zero when the material is locally stretched. Given that {*x*^*i*^} are convected coordinates, *e*_*i*_=F(*E*_*i*_). This allows us to obtain the component expressions C_*ij*_=F(*E*_*i*_)⋅F(*E*_*j*_)=g_*ij*_ and $E_{ij}=\frac{1}{2}(g_{ij}-G_{ij})$. Hence, we see that using convected coordinates, the metric can be used to encode stretching information. However, our definition is coordinate free.

In three-dimensional elasticity, the strain tensor is sufficient to characterize linear constitutive theory. When dealing with shells we must also consider the bending of the shell, or more precisely, the change of curvature from the reference to the spatial configuration. Hence, we define the *change of curvature tensor* $H(Y)=\bar{F}bF(Y)-B(Y)$. Using convected coordinates, we obtain the component expression H_*ij*_=b_*ij*_−B_*ij*_.

We are also interested in the *strain rate*, and to this end we consider the rate of change of a tangent vector as it is convected with the surface, ∂F(*Y*)/∂*t*=∂^2^*ϕ*_*t*_(*X*)/∂*t*∂*η*=∂/∂*η*(∂*ϕ*_*t*_(*X*)/∂*t*)=F(*Y*)⋅∂*v*=*Y* ⋅∂*V* , where *v* and *V* are the velocities referred to the spatial and reference configurations, respectively (figure 1). Using the fact that *e*_3_ is always normal to {*e*_*i*_}, we can write ∂*e*_3_/∂*t*=−*e*_3_⋅(∂*e*_*i*_/∂*t*)*e*^*i*^=−*e*_3_⋅(*e*_*i*_⋅∂*v*)*e*^*i*^=−*v*_3|*i*_*e*^*i*^, where *v*_*a*|*i*_ is defined by *v*_*a*|*i*_=*e*_*a*_⋅(*e*_*i*_⋅∂*v*). Combining these we can now construct a function that returns the rate of change of a vector, that need not be tangential to *S*, as it is convected with the motion *ϕ*_*t*_. We denote this function l(*y*)=∂*y*/∂*t*=*y*⋅∂*v*+*y*⋅*e*_3_(∂*e*_3_/∂*t*) (note that *y* need not be tangential to *S* in this expression). It is useful to decompose this into its symmetric and antisymmetric parts $n(y)=\frac{1}{2}(l(y)+\bar{l}(y))$ and $w(y)=\frac{1}{2}(l(y)-\bar{l}(y))$. After some manipulation, the components of n and w are given, in terms of convected coordinates, by $n_{ij}=\frac{1}{2}(v_{i|j}+v_{j|i})$, n_3*i*_=n_*i*3_=n_33_=0, $w_{ij}=\frac{1}{2}(v_{i|j}-v_{j|i})$, w_3*i*_=−w_*i*3_=*v*_3|*i*_ and w_33_=0.

The symmetric tensor n(*y*) is closely related to E. We define the *rate of change of the strain tensor* E with time by $\dot{E}(Y)=\partial E(Y)/\partial t$. The components of this tensor are given by ${\dot{E}}_{ij}=n_{ij}$, and hence we see that $\dot{E}(Y)=\bar{F}nF(Y)$. This will be important in constitutive theory.

The *rate of change of the change of curvature tensor* $\dot{H}(Y)=\partial H(Y)/\partial t$ can be expressed in component form as ${\dot{H}}_{ij}=\partial l_{3j}/\partial x^{i}-\gamma_{ij}^{k}l_{3k}-\gamma_{i3}^{a}l_{aj}=l_{3j|i}=e_{3}\cdot (e_{i}\cdot \dot{\partial }\dot{l}(e_{j}))$ (for details see appendix A). We see from this that the rate of change of H with time is the *e*_3_ component of the second spatial derivative of velocity. Note that both $\dot{E}$ and $\dot{H}$ are symmetric.

w is an antisymmetric function mapping vectors on *S* into vectors in $E^{3}$. Hence, it has a single characteristic eigenbivector *ω* such that w(*y*)=*y*⋅*ω*, which we can extract as $\omega =\frac{1}{2}e^{a}\wedge w(e_{a})$ [1], §3.4. Defined in this way *ω* is the local *angular velocity* of the shell material, represented as a bivector. The vector representation of angular velocity is given by −*I*_3_*ω*. If we consider *e*_3_ being convected with a material point on the surface, then the fact that it is defined to be a unit vector allows us to use *ω* to write ∂*e*_3_/∂*t*=*e*_3_⋅*ω*. We need a representation of angular velocity in shell theory because it is not possible to assume, as it is in three-dimensional elasticity, that couple-stresses are negligible. The bivector representation of angular velocity allows for a much more physical representation of the governing laws of shells than that suggested by Naghdi [6], who requires the use of a rotated angular velocity with components normal to the shell removed.

## 4. Stress

We consider an arbitrary region of the shell defined by *U*⊂*B*, which under the motion *ϕ*_*t*_ moves to *ϕ*_*t*_(*U*)⊂*S*. In continuum mechanics, it is standard to assume that all the forces on the region *U* can be described by either body forces or boundary forces, to which we must add body and boundary moments in shell theory. *Body forces* are expressed in terms of the body force per unit mass *b*(*x*,*t*). The force acting on the region *U* owing to body forces is given by

4.1 $$\int_{\phi_{t}(U)}\rho b|dx|=\int_{U}\rho b\det F|dX|,$$

where *ρ* is the mass per unit area of the shell, *b*(*X*,*t*)=*b*(*ϕ*_*t*_(*X*),*t*) and *dx*,*dX* are directed volume elements on the spatial and reference configurations. Directed integration theory is introduced in [3], §6.4. In shell theory, we must also consider *body moments*. We define the body moment per unit mass *c* such that the moment acting on the region *U* owing to body moments is given by

4.2 $$\int_{\phi_{t}(U)}\rho c|dx|=\int_{U}\rho c\det F|dX|.$$

Next, we consider boundary forces and moments. We denote a small portion of the boundary ∂*ϕ*_*t*_(*U*) by Δ*s*, with normal vector *n*. We assume that the material on the outside of Δ*s* exerts a force Δ*f*, and moment Δ*m*, on the material inside. The stress principle of Euler and Cauchy, adapted for a shell, states,

as the length Δ*s* tends to zero, the ratios Δ*f*/Δ*s* and Δ*m*/Δ*s* tend to definite limits. Moreover, if two paths passing through a point *x* have the same normal *n*, then Δ*f*/Δ*s* and Δ*m*/Δ*s* tend to the same value for both of these paths [12].

Using arguments outlined by, among others, [12], we can show that the limits described in this principle can be expressed as linear functions of the normal vector *n* at each point *x*∈*S*, given a particular time. This allows us to define the *Cauchy stress tensor* *σ*(*n*) and the *couple-stress tensor* m(*n*). We can then write the force on a portion of the shell owing to boundary forces, and the moment on a portion of the shell owing to couple-stresses as

4.3 $$\int_{\partial \phi_{t}(U)}\sigma (n)|ds|=\int_{\phi_{t}(U)}\dot{\sigma }(\dot{\partial })|dx|,\;\int_{\partial \phi_{t}(U)}m(n)|ds|=\int_{\phi_{t}(U)}\dot{m}(\dot{\partial })|dx|,$$

where d*s* is a directed boundary element, related to the normal vector by *n*|d*s*|=d*si*^−1^.

*σ* and m are both tensors on the spatial configuration. We wish to express balance laws on the reference configuration, so we construct the *first Piola–Kirchhoff stress tensor* T, and the *first reference couple-stress tensor* M, by $T(N)=\det F\;\sigma {\bar{F}}^{-1}(N)$ and $M(N)=\det F\;m{\bar{F}}^{-1}(N)$. Using these we can write

4.4 $$\int_{\partial \phi_{t}(U)}\sigma (n)|ds|=\int_{\partial U}T(N)|dS|,\;\int_{\partial \phi_{t}(U)}m(n)|ds|=\int_{\partial U}M(N)|dS|,$$

where d*S* is a directed boundary element on the reference configuration, related to the normal vector by *N*|d*S*|=d*SI*^−1^. For reasons that become clearer when considering conservation of energy and constitutive law, we also define the *second Piola–Kirchhoff stress tensor* S(*N*)=F^−1^T(*N*) and the *second reference couple-stress tensor* N(*N*)=F^−1^M(*N*).

The domain of *σ* is vectors tangential to *S*, but its range is $E^{3}$, and similarly the domains of T and S are vectors tangential to *B*, while their ranges are $E^{3}$. m is not vector valued, but bivector valued, as it represents a moment. Its domain is vectors tangential to *S*, and its range is the space of all bivectors in $E^{3}$. However, we know that the moments represented by m are because of the stress distribution through the thickness of the shell, and this means that its range is more restricted. More precisely, we can say that m^(1,2)*i*^=(*e*^2^∧*e*^1^)⋅m(*e*^*i*^)=0. Similarly, we assume that *c* is due only to shear stresses acting on the upper and lower surfaces of the shell, meaning that its *e*_1_∧*e*_2_ component is zero. Given the use of convected coordinates, the following coordinate expressions hold:

4.5 $$\begin{array}{rl} e^{a}\cdot T(E^{i}) & =T^{ai}=E^{a}\cdot S(E^{i})=S^{ai}\\ \;\text{and}\;e^{I}\cdot M(E^{i}) & =M^{Ii}=E^{I}\cdot N(E^{i})=N^{Ii}. \end{array}\}$$

It is convenient to define the *modified first reference couple stress tensor* **M**(*N*)=M(*N*)⋅*e*_3_ and the *modified second reference couple stress tensor* **N**(*N*)=N(*N*)⋅*E*_3_. These are vector valued, rather than bivector valued. The restrictions on the range of m imply that the range of **M** is the tangent space of *S*, and the range of **N** is the tangent space of *B*. The symmetry of the three-dimensional Cauchy stress tensor implies that **N** is symmetric in the plane tangent to *B*. These are convenient when expressing conservation of angular momentum and constitutive laws. Note that **M** and **N** are not the physical vector representations of the torque. Their natural emergence in conservation of angular momentum and energy (see §5) explains why [6] was able to make use of an apparently unphysical rotated angular velocity in his formulation, and also justifies the rather strange definition of the vector components of the couple stress given in [5], §1.6.1,eq.1.113. **M** and **N** satisfy the following coordinate expressions:

4.6 $$\begin{array}{rl} e^{1}\cdot M(E^{i}) & =M^{1i}=M^{(1,3)i}=N^{(1,3)i}=N^{1i}=E^{1}\cdot N(E^{i})\\ \;\text{and}\;e^{2}\cdot M(E^{i}) & =M^{2i}=M^{(2,3)i}=N^{(2,3)i}=N^{2i}=E^{2}\cdot N(E^{i}). \end{array}\}$$

## 5. Balance laws

We write each balance law as an integral equation expressed on the spatial configuration, and a local equation of motion expressed on the reference configuration. We are able to express all of these in component free form, which is a common advantage of using GA.

*Mass*
  5.1 $$\frac{d}{dt}\int_{\phi_{t}(U)}\rho |dx|=0$$
  and
  5.2 $$\frac{\partial }{\partial t}(\rho \det F)=0.$$
  Using this we can define the time independent density $\rho_{0}=\rho \det F$.
*Momentum*
  5.3 $$\frac{d}{dt}\int_{\phi_{t}(U)}\rho v|dx|=\int_{\partial \phi_{t}(U)}\sigma (n)|ds|+\int_{\phi_{t}(U)}\rho b|dx|$$
  and
  5.4 $$\rho_{0}\frac{\partial V}{\partial t}=\dot{T}(\dot{\partial })+\rho_{0}b.$$
*Angular momentum*
  5.5 $$\frac{d}{dt}\int_{\phi_{t}(U)}\rho x\wedge v|dx|=\int_{\partial \phi_{t}(U)}x\wedge \sigma (n)+m(n)|ds|+\int_{\phi_{t}(U)}\rho x\wedge b+\rho c|dx|$$
  and
  5.6 $$\dot{\phi_{t}(X)}\wedge T(\dot{\partial })+\dot{M}(\dot{\partial })+\rho_{0}c=0.$$
  The algebraic manipulations necessary to achieve this expression are given in appendix C. We can split this expression into its *e*_1_∧*e*_3_, *e*_2_∧*e*_3_ and *e*_1_∧*e*_2_ components. These components involve taking the divergence of a bivector valued function, which is outlined by Hestenes & Sobczyk [1]. However, using the modified first couple-stress tensor, these components can be written in a more familiar form:
  5.7 $$\begin{array}{rl} & T^{3i}+\frac{\partial M^{ij}}{\partial X^{j}}+M^{kj}\gamma_{jk}^{i}+M^{ik}\Gamma_{jk}^{j}+\rho c^{i}=0\\ \;\text{and}\; & T^{21}-T^{12}+M^{2i}{b^{1}}_{i}-M^{1i}{b^{2}}_{i}=0, \end{array}\}$$
  where, for convenience, we have defined *c*^1^=*c*⋅(*e*^3^∧*e*^1^)=*c*^(1,3)^ and *c*^2^=*c*⋅(*e*^3^∧*e*^2^)=*c*^(2,3)^. The bivector versions of these expressions are equally valid, and easier to interpret physically, but less familiar because they involve bivector components. To obtain more familiar expressions, we need to use modified tensors such as **M**, whose physical meaning is less immediately obvious. The use of bivectors to represent angular velocities and torques has illuminated why it was necessary for [6] to use apparently unphysical quantities to develop his shell theory.
  Conservation of angular momentum has two major implications. The first, from the *e*_*i*_∧*e*_3_ components of the expression, is that stresses normal to the tangent plane of the surface are determined if the couple-stress and body moment are known. This means that we do not need a constitutive law for these components of the stress, we only need constitutive laws for the components of stress within the plane of the shell, and for the couple stress. The second implication, from the *e*_1_∧*e*_2_ component, is that the *modified second Piola–Kirchhoff stress* $\tilde{S}(Y)=S(Y)-F^{-1}bF\bar{N}(Y)$ is symmetric in the tangent space of the reference configuration. This is important when considering conservation of energy and in constitutive theory.
Energy
In this article, we assume isothermal elasticity. It is uncomplicated to include thermal effects, simply requiring the inclusion of the second law of thermodynamics and additional constitutive laws, but this addition does not contribute to our aim here of introducing GA to shell theory for the first time, so is not included. Conservation of energy is therefore given by
  5.8 $$\begin{array}{rl} \frac{d}{dt}\int_{\phi_{t}(U)}\rho \left(e+\frac{v^{2}}{2}\right)|dx| & =\int_{\phi_{t}(U)}\rho (v\cdot b-\omega \cdot c)|dx|\\ & \;+\int_{\partial \phi_{t}(U)}v\cdot \sigma (n)-\omega \cdot m(n)|ds|, \end{array}$$
  where *e*(*x*,*t*) is the internal energy per unit mass. The negative signs before the moment terms is consistent with the use of bivectors to represent moments and angular velocities (see appendix B). After some algebraic manipulation (see appendix D), on the reference configuration we obtain
  5.9 $$\rho_{0}\frac{\partial E}{\partial t}=\mathrm{tr}(\tilde{S}\dot{E})+\mathrm{tr}(N\dot{H}),$$
  where *E*(*X*,*t*)=*e*(*ϕ*_*t*_(*X*),*t*) (which is not the same as E, the Green–Lagrange strain tensor). Note the appearance of the modified second Piola–Kirchhoff stress, and the modified second couple stress tensor. We know the first of these is symmetric in the tangent space of *B* from conservation of angular momentum, and the second must be assumed symmetric in order to obtain a determinate theory (this assumption was first proposed in [6], §15). This allows us to use this expression to derive the constitutive laws given in §6.

## 6. Constitutive theory

Our basic constitutive assumption is that *E* is a function of the local values of the tensors E and H. Applying the chain rule to equation (5.9), and noting that the equation is valid for arbitrary deformations, we obtain the constitutive relations:

6.1 $$S(Y)-F^{-1}bFN(Y)=\rho_{0}\frac{\partial E}{\partial E(Y)},\;N(Y)=\rho_{0}\frac{\partial E}{\partial H(Y)}.$$

For an introduction to tensor derivatives, see [3], §11.1.2.

Koiter [10] proposes the following form for *ρ*_0_*E*, which can be regarded as the application of the Saint Venant–Kirchhoff material to shells:

6.2 $$\rho_{0}E=\frac{E_{y}h}{2(1-\nu^{2})}((1-\nu )\;\mathrm{tr}(E^{2})+\nu \;\mathrm{tr}{\left(E\right)}^{2})+\frac{E_{y}h^{3}}{24(1-\nu^{2})}((1-\nu )\;\mathrm{tr}(H^{2})+\nu \;\mathrm{tr}{\left(H\right)}^{2}),$$

where *E*_*y*_ is Young’s modulus, *ν* is Poisson’s ratio and *h* is the thickness of the shell. From this we obtain the following relationships:

6.3 $$\begin{array}{rl} S(Y)-F^{-1}bFN(Y) & =\frac{E_{y}h}{1-\nu^{2}}((1-\nu )E(Y)+\nu \;\mathrm{tr}(E)Y)\\ \;\text{and}\;N(Y) & =\frac{E_{y}h}{12(1-\nu^{2})}((1-\nu )H(Y)+\nu \;\mathrm{tr}(H)Y). \end{array}\}$$

Note that this only provides the part of S that is tangential to *B*. The non-tangential part (S^3*i*^) is found using conservation of angular momentum.

There is a fundamental contradiction in arriving at the results presented here. To arrive at the presented form of *ρ*_0_*E* shown, we must make the following assumptions:

— the midsurface in the reference configuration remains the midsurface under the motion;
— a material line that is normal to the midsurface in the reference configuration remains normal to the midsurface;
— the shell thickness (measured normal to the midsurface) is constant over the surface and does not change with time;
— the first and second moments of the density relative to the midsurface are zero;
— the shell thickness is small compared with its principal radii of curvature;
— strains within the shell are small; and
— normal stress in the shell is negligible.

When applied to Hooke’s law in three dimensions, these assumptions imply that the *e*_3_ component of *σ* is zero, but we know that in shell theory these components are required for conservation of angular momentum. This basic contradiction remains unresolved.

## 7. Linearization

We define the displacement *U*(*X*,*t*)=*ϕ*_*t*_(*X*)−*X*, and we assume that it takes the form *U*=*U*_0_+*ϵU*′, where *ϵ* is small. Neglecting terms of $O(\epsilon^{2})$ we obtain

7.1 $$\begin{array}{rl} F(Y) & =Y+Y\cdot \partial U=Y+Y\cdot \partial U_{0}+\epsilon Y\cdot \partial U^{\prime }=F_{0}(Y)+\epsilon F^{\prime }(Y),\\ \det F & =\det F_{0}+\epsilon \det F_{0}\;\mathrm{tr}(F_{0}^{-1}F^{\prime }),\\ F^{-1}(Y) & =F_{0}^{-1}(Y)-\epsilon F_{0}^{-1}F^{\prime }F_{0}^{-1}(Y)\\ \;\text{and}\;\;\det F^{-1} & =\det F_{0}^{-1}+\epsilon \det F_{0}^{-1}\;\mathrm{tr}(F^{\prime }F_{0}^{-1}). \end{array}\}$$

The Green–Lagrange strain tensor can then be written as

7.2 $$2E(Y)={\bar{F}}_{0}F_{0}(Y)-Y+\epsilon ({\bar{F}}_{0}F^{\prime }(Y)+{\bar{F}}^{\prime }F_{0}(Y))=2E_{0}(Y)+2\epsilon E^{\prime }(Y).$$

We also need to write the change of curvature tensor in its perturbed form. To do this, we first express the convected basis vectors {*e*_*a*_} as

7.3 $$\begin{array}{rl} e_{i} & =F_{0}(E_{i})+\epsilon F^{\prime }(E_{i}),\\ e_{3} & =\frac{\det F_{0}^{-1}}{V}F_{0}(E_{1})\times F_{0}(E_{2})+\epsilon \frac{\det F_{0}^{-1}}{V}(F^{\prime }(E_{1})\times F_{0}(E_{2})\\ & \;+F_{0}(E_{1})\times F^{\prime }(E_{2})-\mathrm{tr}(F^{\prime }F_{0}^{-1})F_{0}(E_{1})\times F_{0}(E_{2}))\\ & =e_{30}+\epsilon e_{3}^{\prime }, \end{array}$$

where × is the vector cross product, defined by *a*×*b*=−*I*_3_*a*∧*b*. This allows us to write $\bar{F}bF$ as

7.4 $$\begin{array}{rl} \bar{F}bF(Y) & =Y^{i}E^{j}e_{30}\cdot \partial_{i}F_{0}(E_{j})+\epsilon Y^{i}E^{j}(e_{30}\cdot \partial_{i}F^{\prime }(E_{j})+e_{3}^{\prime }\cdot \partial_{i}F_{0}(E_{j}))\\ & ={\left(\bar{F}bF\right)}_{0}(Y)+\epsilon {\left(\bar{F}bF\right)}^{\prime }(Y), \end{array}$$

which in turn allows us to express H as

7.5 $$H(Y)={\left(\bar{F}bF\right)}_{0}(Y)-B(y)+\epsilon {\left(\bar{F}bF\right)}^{\prime }(Y)=H_{0}(Y)+\epsilon H^{\prime }(Y).$$

We can now write the modified second reference couple stress tensor **N** as

7.6 $$\begin{array}{rl} N(y) & =\frac{Eh^{3}}{12(1-\nu^{2})}((1-\nu )H_{0}(y)+\nu \;\mathrm{tr}(H_{0})y)+\epsilon \frac{Eh^{3}}{12(1-\nu^{2})}((1-\nu )H^{\prime }(y)+\nu \;\mathrm{tr}(H^{\prime })y)\\ & =N_{0}(y)+\epsilon N^{\prime }(y). \end{array}$$

To express the second Piola–Kirchhoff stress tensor, we first need to express F^−1^bF(*Y*):

7.7 $$\begin{array}{rl} F^{-1}bF(y) & =Y^{i}E_{j}e_{30}\cdot \partial_{i}{\bar{F}}_{0}^{-1}(E^{j})+\epsilon Y^{i}E_{j}(-e_{30}\cdot \partial_{i}{\bar{F}}_{0}^{-1}{\bar{F}}^{\prime }{\bar{F}}_{0}^{-1}(E^{j})+e_{3}^{\prime }\cdot \partial_{i}{\bar{F}}_{0}^{-1}(E^{j}))\\ & ={\left(F^{-1}bF\right)}_{0}(y)+\epsilon {\left(F^{-1}bF\right)}^{\prime }(y). \end{array}$$

This allows us to write S and T as

7.8 $$\begin{array}{rl} S(Y) & \approx \frac{Eh}{1-\nu^{2}}((1-\nu )E_{0}(Y)+\nu \;\mathrm{tr}(E_{0})Y)+\epsilon \frac{Eh}{1-\nu^{2}}((1-\nu )E^{\prime }(Y)+\nu \;\mathrm{tr}(E^{\prime })Y)\\ & \;+{\left(F^{-1}bF\right)}_{0}N_{0}(Y)+\epsilon ({\left(F^{-1}bF\right)}_{0}N^{\prime }(Y)+{\left(F^{-1}bF\right)}^{\prime }N_{0}(Y))\\ & =S_{0}(Y)+\epsilon S^{\prime }(Y),\\ T(Y) & =F_{0}S_{0}(y)+\epsilon (F_{0}S^{\prime }(y)+F^{\prime }S_{0}(y))\\ & =T_{0}(y)+\epsilon T^{\prime }(y). \end{array}$$

Conservation of mass can be expressed as

7.9 $$\frac{\partial }{\partial t}(\rho \det F)=\frac{\partial }{\partial t}(\rho (\det F_{0}+\epsilon \det F_{0}\;\mathrm{tr}(F_{0}^{-1}F^{\prime })))=0.$$

We define $\rho_{0}=\rho \det F_{0}$ and $\rho^{\prime }=\rho \det F_{0}\mathrm{tr}(F_{0}^{-1}F^{\prime })$ (note the adjustment of the definition of *ρ*_0_). We assume that the initial displacement *U*_0_ satisfies the governing equations separately, so both *ρ*_0_ and *ρ*′ are independent of time. Conservation of momentum can be expressed as

7.10 $$(\rho_{0}+\epsilon \rho^{\prime })\frac{\partial^{2}}{\partial t^{2}}(U_{0}+\epsilon U^{\prime })={\dot{T}}_{0}(\dot{\partial })+\epsilon {\dot{T}}^{\prime }(\dot{\partial })+(\rho_{0}+\epsilon \rho^{\prime })b.$$

We denote the body force acting on the body in its initial deformed state (defined by *U*_0_) by *b*_0_, and then decompose *b* as *b*=*b*_0_+*ϵb*′. This includes the assumption that the additional body force acting on the body after the perturbation *ϵU*′ is small. Subtracting conservation of momentum for the initial deformation *U*_0_, we obtain

7.11 $$\rho^{\prime }\frac{\partial^{2}U_{0}}{\partial t^{2}}+\rho_{0}\frac{\partial^{2}U^{\prime }}{\partial t^{2}}={\dot{T}}^{\prime }(\dot{\partial })+\rho_{0}b^{\prime }+\rho^{\prime }b_{0}.$$

Usually we assume that *U*_0_ is time independent, meaning that we obtain

7.12 $$\rho_{0}\frac{\partial^{2}U^{\prime }}{\partial t^{2}}={\dot{T}}^{\prime }(\dot{\partial })+\rho_{0}b^{\prime }+\rho^{\prime }b_{0}.$$

We can write M as

7.13 $$\begin{array}{rl} M(Y) & =FN(Y)\wedge e_{3}\\ & =F_{0}N_{0}(Y)\wedge e_{30}+\epsilon (F^{\prime }N_{0}(Y)\wedge e_{30}+F_{0}N^{\prime }(Y)\wedge e_{30}+F_{0}N_{0}(Y)\wedge e_{3}^{\prime })\\ & =M_{0}(y)+\epsilon M^{\prime }(y). \end{array}$$

If, as with *b*, we assume that *c* can be decomposed as *c*=*c*_0_+*ϵc*′, then this allows us to write the perturbed part of conservation of angular momentum as

7.14 $$F_{0}(E_{i})\wedge T^{\prime }(E^{i})+F^{\prime }(E_{i})\wedge T_{0}(E^{i})+{\dot{M}}^{\prime }(\dot{\partial })+\rho_{0}c^{\prime }+\rho^{\prime }c_{0}=0.$$

### 7.1. Small displacements

If we assume *U*_0_=0 (or that it is constant), then we obtain the following simplifications:

7.15 $$\begin{array}{rl} F(Y) & =Y+\epsilon Y\cdot \partial U^{\prime }=Y+\epsilon F^{\prime }(Y),\\ F^{-1}(Y) & =Y-\epsilon F^{\prime }(Y),\\ 2E(y) & =\epsilon (F^{\prime }(Y)+{\bar{F}}^{\prime }(Y))=Y\cdot \partial U+\dot{\partial }(Y\cdot \dot{U}),\\ \det F & =1+\epsilon \;\mathrm{tr}(F^{\prime })=1+\epsilon \partial \cdot U^{\prime },\\ \det F^{-1} & =1-\epsilon \;\mathrm{tr}(F^{\prime })=1-\epsilon \partial \cdot U^{\prime },\\ e_{i} & =E_{i}+\epsilon f^{\prime }(E_{i})=E_{i}+\epsilon E_{i}\cdot \partial U^{\prime },\\ e^{i} & =E^{i}-\epsilon {\bar{F}}^{\prime }(E^{i})=E^{i}-\epsilon \dot{\partial }(E^{i}\cdot {\dot{U}}^{\prime })\\ \;\text{and}\;e_{3} & =E_{3}+\epsilon \left(\frac{1}{V}(E_{1}\cdot \partial U^{\prime })\times E_{2}+\frac{1}{V}E_{1}\times (E_{2}\cdot \partial U^{\prime })-(\partial \cdot u^{\prime })E_{3}\right). \end{array}\}$$

Following the method of Ciarlet [7], and using the coordinate independent notation of GA, the change of curvature tensor takes the form

7.16 $$H(Y)=\epsilon E^{j}((E_{j}\cdot \dot{\partial }{\dot{F}}^{\prime }(Y))\cdot E_{3}).$$

From this, it is clear how the linearized change of curvature tensor is closely related to the *E*_3_ components of the second derivative of the displacement field *U*′.

Using these results and applying the formulae of the previous section we have, S_0_(*Y*)=0, T_0_(*Y*)=0, **N**_0_(*Y*)=0, M_0_(*Y*)=0, and

7.17 $$\begin{array}{rl} N^{\prime }(Y) & =\frac{E_{y}h^{3}}{12(1-\nu^{2})}((1-\nu )H^{\prime }(Y)+\nu \;\mathrm{tr}(H^{\prime })Y),\\ S(Y) & =\frac{E_{y}h}{1-\nu^{2}}((1-\nu )E^{\prime }(Y)+\nu \;\mathrm{tr}(E^{\prime })Y)+\frac{E_{y}h^{3}}{12(1-\nu^{2})}(\left(1-\nu )BH^{\prime }(Y)+\nu \;\mathrm{tr}(H^{\prime })B(Y\right))\\ \;\text{and}\;M^{\prime }(Y) & =N^{\prime }(Y)\wedge E_{3}. \end{array}\}$$

Conservation of momentum and angular momentum can be written as

7.18 $$\rho_{0}\frac{\partial^{2}U^{\prime }}{\partial t^{2}}={\dot{S}}^{\prime }(\dot{\partial })+\rho_{0}b^{\prime }$$

and

7.19 $$E_{i}\wedge S^{\prime }(E^{i})+{\dot{M}}^{\prime }(\dot{\partial })+\rho^{\prime }c_{0}=0.$$

It is worth pointing out an anomaly at this point, which is made clearer by the use of GA. Much of the previous work done on linearized shell theory (summarized by Leissa [5], with one of the more rigorous derivations provided by Vlasov [13,14]) uses a rather strange coordinate system, which does not aid comprehension. Coordinates are chosen such that the lines *X*^*i*^=*constant* define lines of principal curvature on the reference configuration. This allows the components of several tensors to be expressed more simply, as the basis vectors are orthogonal, and are eigenvectors of B. However, the coordinate system is not constrained to be orthonormal, meaning that the reciprocal frame and frame do not coincide (though *E*^*i*^ is parallel to *E*_*i*_). This is not a problem in GA, as we can use an arbitrary coordinate system, but the solution adopted by many authors is to create a new normalized frame $\left\{{\hat{E}}_{i}=E_{i}/\left|E_{i}\right|\right\}$. Differentiation is performed with respect to the coordinates {*X*^*i*^}, but tensor and vector components are expressed relative to the frame $\left\{{\hat{E}}_{i}\right\}$. This adds considerable complication to the expressions for strain and change of curvature, which, through the use of GA, we have simplified.

### 7.2. Uniaxial strain of a cylinder

We now consider the case in which {*E*_*i*_} forms an orthonormal basis, and are also the eigenvectors of B. In this case, we have $\Gamma_{jk}^{i}=0$ and

7.20 $$B_{11}=\Gamma_{11}^{3}=-\Gamma_{13}^{1}=C_{1},\;B_{12}=\Gamma_{12}^{3}=-\Gamma_{13}^{2}=0,\;B_{22}=\Gamma_{22}^{3}=-\Gamma_{23}^{2}=C_{2}.$$

Moreover, we take *C*_1_=0 and *C*_2_=*C*. We take the background deformation to be uniaxial strain such that *U*_0_=*εX*^1^*E*_1_. *X*^1^ is the axial distance along the cylindrical shell, and *X*^2^ is the azimuthal distance around the circumference. We can write F_0_ as F_0_(*Y*)=*Y* +*ε*(*Y* ⋅*E*_1_)*E*_1_, but *E*_1_ is a basis vector specific to the tangent space of the reference configuration. For clarity, we therefore define a unit vector aligned with the axis of symmetry of the cylindrical shell ***e***, which is defined everywhere in $E^{3}$. On the reference configuration ***e***=*E*_1_. Using this we write:

7.21 $$\begin{array}{rl} F_{0}(Y) & =Y+\varepsilon (Y\cdot e)e,\\ {\bar{F}}_{0}(y) & =y+\varepsilon (y\cdot e)e,\\ f_{0}^{-1}(y) & =y-\frac{\varepsilon }{\lambda }(y\cdot e)e,\\ {\bar{F}}_{0}^{-1}(Y) & =Y-\frac{\varepsilon }{\lambda }(Y\cdot e)e\\ \;\text{and}\;\det F_{0} & =1+\varepsilon =\lambda , \end{array}\}$$

where we have also defined λ=1+*ε*. Given that the frame *E*_*i*_ is orthonormal, we do not need to distinguish sub- and super-script indices. Hence, we can obtain the following expression for the components of the tensors derived in previous sections:

7.22 $$\begin{array}{rl} & \begin{array}{rl} & {\left(E_{0}\right)}_{11}=\frac{1}{2}\varepsilon^{2}+\varepsilon ,\;{\left(E_{0}\right)}_{12}={\left(E_{0}\right)}_{21}=0,\;{\left(E_{0}\right)}_{22}=0,\\ & {\left(E^{\prime }\right)}_{11}=(1+\varepsilon )\partial_{1}U_{1}^{\prime },\;{\left(E^{\prime }\right)}_{22}=\partial_{2}U_{2}^{\prime }-CU_{3}^{\prime },\\ & {\left(E^{\prime }\right)}_{12}={\left(E^{\prime }\right)}_{21}=\frac{1}{2}(\partial_{1}U_{2}^{\prime }+\partial_{2}U_{1}^{\prime })+\frac{\varepsilon }{2}\partial_{2}U_{1}^{\prime }. \end{array}\} \end{array}$$

7.23 $$\begin{array}{rl} & \begin{array}{rl} & {\left(H_{0}\right)}_{ij}=0,\\ & H_{11}^{\prime }=\partial_{1}\partial_{1}U_{3}^{\prime },\;H_{22}^{\prime }=\partial_{2}\partial_{2}U_{3}^{\prime }+2C\partial_{2}U_{2}^{\prime }-C^{2}U_{3}^{\prime },\\ \;\text{and}\; & H_{12}^{\prime }=H_{21}^{\prime }=\partial_{1}\partial_{2}U_{3}^{\prime }+C\partial_{1}U_{2}^{\prime }. \end{array}\} \end{array}$$

7.24 $$\begin{array}{rl} & \begin{array}{rl} & {\left(N_{0}\right)}_{ij}=0,\\ & N_{11}^{\prime }=\frac{E_{y}h^{3}}{12(1-\nu^{2})}(H_{11}^{\prime }+\nu H_{22}^{\prime }),\\ & N_{22}^{\prime }=\frac{E_{y}h^{3}}{12(1-\nu^{2})}(H_{22}^{\prime }+\nu H_{11}^{\prime })\\ \;\text{and}\; & N_{12}^{\prime }=N_{21}^{\prime }=\frac{E_{y}h^{3}}{12(1+\nu )}H_{12}^{\prime }. \end{array}\} \end{array}$$

7.25 $$\begin{array}{rl} & \begin{array}{rl} & {\left(S_{0}\right)}_{11}=\frac{E_{y}h}{1-\nu^{2}}{\left(E_{0}\right)}_{11},\;{\left(S_{0}\right)}_{22}=\frac{E_{y}h}{1-\nu^{2}}\nu {\left(E_{0}\right)}_{11}\\ \;\text{and}\; & {\left(S_{0}\right)}_{12}={\left(S_{0}\right)}_{21}=0. \end{array}\} \end{array}$$

7.26 $$\begin{array}{rl} & \begin{array}{rl} & S_{11}^{\prime }=\frac{E_{y}h}{1-\nu^{2}}(E_{11}^{\prime }+\nu E_{22}^{\prime })+CN_{22}^{\prime },\;S_{22}^{\prime }=\frac{E_{y}h}{1-\nu^{2}}(E_{22}^{\prime }+\nu E_{11}^{\prime })\\ \;\text{and}\; & S_{12}^{\prime }=\frac{E_{y}h}{1+\nu }E_{12}^{\prime },\;S_{21}^{\prime }=\frac{E_{y}h}{1+\nu }E_{21}^{\prime }+CN_{21}^{\prime }. \end{array}\} \end{array}$$

This demonstrates the application of linearized shell theory to a situation where there is prior strain.

## 8. Conclusion

The elastic theory of shells has been advanced using GA, providing clarifications and some new developments. We have provided a lucid, geometric interpretation of $\det(G_{ij})=V^{2}$, and clarified the difference between the coordinate definition $\det(G_{ij})$ and the coordinate free definition of the determinant of G. As has been the case in other areas, GA has allowed a coordinate free representation of balance laws and constitutive laws, which makes physical interpretation clearer, while also providing the tools to easily express these equations in terms of arbitrary coordinate systems for practical purposes. The role of moments and angular velocity, and the apparent use by previous authors of an unphysical angular velocity, has been clarified through the use of a bivector representation. We hope that this early work using GA will allow the powerful encoding of rotations by GA, using rotors, to be used in a similar way as has been done for rods [4]. In linearized theory clarification of confusing previous coordinate conventions has been provided, and the introduction of prior strain into the linearized theory of shells has been made possible.

## Appendix A. The rate of change of curvature

The components of $\dot{H}$ are given by ${\dot{H}}_{ij}=\partial H_{ij}/\partial t$. The component expression H_*ij*_=b_*ij*_−B_*ij*_ has already been derived, and B does not change with time, so we can write:

A 1 $$\begin{array}{rl} {\dot{H}}_{ij}=\frac{\partial H_{ij}}{\partial t}=\frac{\partial b_{ij}}{\partial t} & =\frac{\partial }{\partial t}\left(e_{3}\cdot \frac{\partial e_{j}}{\partial x^{i}}\right)\\ & =\frac{\partial e_{3}}{\partial t}\cdot \frac{\partial e_{j}}{\partial x^{i}}+e_{3}\cdot \frac{\partial }{\partial x^{i}}\frac{\partial e_{j}}{\partial t}\\ & =l_{a3}\gamma_{ij}^{a}+e_{3}\cdot \partial_{i}(l_{aj}e^{a})\\ & =-l_{3a}\gamma_{ij}^{a}+\partial_{i}l_{3j}-l_{aj}\gamma_{i3}^{a}\\ & =\partial_{i}l_{3j}-\gamma_{ij}^{k}l_{3k}-\gamma_{i3}^{a}l_{aj}. \end{array}$$

Here, we have used the fact that l_3*i*_=−l_*i*3_, and l_33_=0. Also, where appropriate, time derivatives have been taken assuming that we are being convected with the surface. The spatial derivative of a function defined on the surface *S* is given by

A 2 $$\begin{array}{rl} y\cdot \dot{\partial }\dot{l}(z) & =y\cdot \partial (l(z))-l(y\cdot \partial z)\\ & =y^{i}\partial_{i}(l_{aj}z^{j}e^{a})-l(y^{i}\partial_{i}(z^{j}e_{j}))\\ & =y^{i}(\partial_{i}(l_{aj})z^{j}e^{a}+l_{aj}\partial_{i}(z^{j})e^{a}+l_{aj}z^{j}\partial_{i}(e^{a})-\partial_{i}(z^{j})l(e_{j})-z^{j}l(\partial_{i}(e_{j})))\\ & =y^{i}z^{j}(\partial_{i}(l_{aj})e^{a}-l_{aj}\gamma_{ib}^{a}e^{b}-\gamma_{ij}^{a}l_{ba}e^{b})\\ & =y^{i}z^{j}(\partial_{i}l_{aj}-\gamma_{ij}^{k}l_{ak}-\gamma_{ia}^{b}l_{bj})e^{a}=y^{i}z^{j}e^{a}l_{aj|i}, \end{array}$$

where we have assumed that l(*e*_3_)=0, and the last equality defines l_*aj*|*i*_. Hence, we see that ${\dot{H}}_{ij}$ is given by

A 3 $${\dot{H}}_{ij}=\partial_{i}l_{3j}-\gamma_{ij}^{k}l_{3k}-\gamma_{i3}^{a}l_{aj}=l_{3j|i}=e_{3}\cdot (e_{i}\cdot \dot{\partial }\dot{l}(e_{j})).$$

## Appendix B. Work done by bivector torque

Let *ω* and *ω*_*v*_ be the bivector and vector representations of the angular velocity of a body, related by *ω*=*I*_3_*ω*_*v*_. If *q*_*v*_ is the vector representation of the torque acting on the body then the rate at which work is done on the body is given by *ω*_*v*_⋅*q*_*v*_. Making use of [3], §4.1.3, we can write this as

B 1 $$\begin{array}{rl} \omega_{v}\cdot q_{v} & =-I_{3}^{2}\omega_{v}\cdot q_{v}\\ & =-\frac{1}{2}I_{3}^{2}(\omega_{v}q_{v}+q_{v}\omega_{v})\\ & =-\frac{1}{2}I_{3}(I_{3}\omega_{v}q_{v}+I_{3}q_{v}\omega_{v})\\ & =-\frac{1}{2}I_{3}(\omega_{v}I_{3}q_{v}+q_{v}I_{3}\omega_{v})\\ & =-\frac{1}{2}(\left(I_{3}\omega_{v})(I_{3}q_{v})+(I_{3}q_{v})(I_{3}\omega_{v}\right))\\ & =-(I_{3}\omega_{v})\cdot (I_{3}q_{v})\\ & =-\omega \cdot (I_{3}q_{v}). \end{array}$$

The bivector representation of torque *q* is related to *q*_*v*_ by *q*=*I*_3_*q*_*v*_ (see [3], §3.1.1), and so the rate of work done by the torque *q* is given by −*ω*⋅*q*.

## Appendix C. Conservation of angular momentum

We can express conservation of angular momentum on the reference configuration as

C 1 $$\begin{array}{rl} \frac{d}{dt}\int_{U}\rho \phi_{t}(X)\wedge V\;\det F|dX| & =\int_{\partial U}\phi_{t}(X)\wedge T(N)+M(N)|dS|\\ & \;+\int_{U}(\rho \phi_{t}(X)\wedge b+\rho c)\det F|dX|. \end{array}$$

Simplifying using conservation of mass, we can express this in local form as

C 2 $$\rho_{0}\phi_{t}(X)\wedge \frac{\partial V}{\partial t}=\phi_{t}(X)\wedge \dot{T}(\dot{\partial })+\dot{\phi_{t}(X)}\wedge T(\dot{\partial })+\dot{M}(\dot{\partial })+\rho_{0}\phi_{t}(X)\wedge b+\rho_{0}c.$$

Using conservation of momentum, this simplifies to

C 3 $$\dot{\phi_{t}(X)}\wedge T(\dot{\partial })+\dot{M}(\dot{\partial })+\rho_{0}c=0.$$

The first term in this expression can be written as

C 4 $$\dot{\phi_{t}(X)}\wedge T(\dot{\partial })=(E_{i}\cdot \partial \phi_{t}(X))\wedge T(E^{i})=F(E_{i})\wedge T(E^{i})=F(E_{i})\wedge FS(E^{i}).$$

We can express $\dot{M}(\dot{\partial })$ as

C 5 $$\begin{array}{rl} \dot{M}(\dot{\partial }) & =\left(\frac{\partial M^{Ii}}{\partial X^{i}}+M^{Ji}\gamma_{iJ}^{I}+M^{Ij}\Gamma_{ij}^{i}\right)e_{I}+M^{Ji}\gamma_{iJ}^{\left(1,2\right)}e_{1}\wedge e_{2}\\ & ={M^{Ii}}_{|i}e_{I}+M^{Ji}\gamma_{iJ}^{\left(1,2\right)}e_{1}\wedge e_{2}. \end{array}$$

Writing conservation of angular momentum in component form (noting that we must use the convected frame {*e*_*i*_}) we obtain

C 6 $$\begin{array}{rl} S^{31}+{M^{(1,3)i}}_{|i}+\rho_{0}c^{\left(1,3\right)} & =0,\\ S^{32}+{M^{(2,3)i}}_{|i}+\rho_{0}c^{\left(2,3\right)} & =0\\ \;\text{and}\;S^{21}-S^{12}+M^{(2,3)i}{b^{1}}_{i}-M^{(1,3)i}{b^{2}}_{i} & =0. \end{array}\}$$

By using the modified first reference couple-stress tensor **M**(*y*)=M(*y*)⋅*e*_3_, we can write the last of these as

C 7 $$S^{21}-S^{12}+M^{2i}{b^{1}}_{i}-M^{1i}{b^{2}}_{i}=0.$$

Alternatively, we can use the modified second reference couple stress tensor **N** to write this as

C 8 $$S^{21}-S^{12}+N^{2i}{b^{1}}_{i}-N^{1i}{b^{2}}_{i}=0.$$

We define the *modified second Piola–Kirchhoff* stress tensor by

C 9 $$\begin{array}{rl} \tilde{S}(y) & =S(y)-F^{-1}bF\bar{N}(y)\\ \;\text{and}\;{\tilde{S}}^{ij} & =S^{ij}-{b^{i}}_{k}{\bar{N}}^{kj}=S^{ij}-{b^{i}}_{k}N^{jk},\;{\tilde{S}}^{3i}=S^{3i}. \end{array}\}$$

Conservation of angular momentum then implies that, ${\tilde{S}}^{21}={\tilde{S}}^{12}$, hence, $\tilde{S}$ is symmetric in the plane of the shell.

## Appendix D. Conservation of energy

Conservation of energy can be expressed on the reference configuration as

D 1 $$\begin{array}{rl} \frac{d}{dt}\int_{U}\rho \left(E+\frac{V^{2}}{2}\right)\det F|dX| & =\int_{U}\rho (V\cdot b-\Omega \cdot c)\det F|dX|\\ & \;+\int_{\partial U}V\cdot T(n)-\Omega \cdot M(n)|dS|, \end{array}$$

where we have defined *Ω* to be the angular velocity referred to the reference configurations *Ω*(*X*,*t*)=*ω*(*ϕ*_*t*_(*X*),*t*). Converting to local form, and making use of conservation of mass and momentum, this can be written as

D 2 $$\rho_{0}\frac{\partial E}{\partial t}=-\rho_{0}\Omega \cdot c+\dot{V}\cdot T(\dot{\partial })-\dot{\Omega }\cdot M(\dot{\partial })-\Omega \cdot \dot{M}(\dot{\partial }).$$

Making use of conservation of angular momentum we obtain

D 3 $$\rho_{0}\frac{\partial E}{\partial t}=\dot{V}\cdot T(\dot{\partial })-\dot{\Omega }\cdot M(\dot{\partial })+\Omega \cdot (\dot{\phi_{t}(X)}\wedge T(\dot{\partial })).$$

S is related to T by T(*y*)=FS(*y*), so we can write conservation of energy as

D 4 $$\rho_{0}\frac{\partial E}{\partial t}=\dot{V}\cdot FS(\dot{\partial })-\dot{\Omega }\cdot M(\dot{\partial })+\Omega \cdot (F(E_{i})\wedge FS(E^{i})).$$

The first term on the r.h.s. in this expression can be expressed as

D 5 $$(E_{i}\cdot \partial V)\cdot FS(E^{i})=(E_{i}\cdot \partial V)\cdot F(E_{a})S^{ai}=(F(E_{i})\cdot \partial v)\cdot F(E_{a})S^{ai}=v_{a|i}S^{ai}.$$

We can write $\Omega \cdot (F(E_{i})\wedge FS(E^{i}))-\dot{\Omega }\cdot M(\dot{\partial })$ as

D 6 $$\begin{array}{rl} \Omega \cdot (F(E_{i})\wedge FS(E^{i}))-\dot{\Omega }\cdot M(\dot{\partial }) & =\omega_{A}e^{A}\cdot (e_{i}\wedge e_{a})S^{ai}-(e_{i}\cdot \partial \omega )\cdot e_{I}M^{Ii}\\ & =\omega_{i}S^{3i}+\omega_{3}(S^{21}-S^{12})-\left(\frac{\partial \omega_{I}}{\partial x^{i}}-\omega_{J}\gamma_{iI}^{J}-\omega_{\left(1,2\right)}\gamma_{iI}^{\left(1,2\right)}\right)M^{Ii}\\ & =\omega_{i}S^{3i}+\omega_{3}(S^{21}-S^{12}+M^{(2,3)i}{b^{1}}_{i}-M^{(1,3)i}{b^{2}}_{i})-\left(\frac{\partial \omega_{I}}{\partial x^{i}}-\omega_{J}\gamma_{iI}^{J}\right)M^{Ii}\\ & =\omega_{i}S^{3i}-\left(\frac{\partial \omega_{I}}{\partial x^{i}}-\omega_{J}\gamma_{iI}^{J}\right)M^{Ii}, \end{array}$$

where, for convenience, we have defined *ω*_1_=*ω*_(1,3)_, *ω*_2_=*ω*_(2,3)_, and *ω*_3_=*ω*_(1,2)_. The components of *ω* can be found using $\omega =\frac{1}{2}e^{a}\wedge w(e_{a})$. The first two components are given by

D 7 $$\omega_{\left(1,3\right)}=v_{3|1},\;\omega_{\left(2,3\right)}=v_{3|2}.$$

Using these expressions for the components of *ω* we obtain

D 8 $$\begin{array}{rl} \Omega \cdot (F(E_{i})\wedge FS(E^{i}))-\dot{\Omega }\cdot M(\dot{\partial }) & =-v_{3|i}s^{3i}+\left(\frac{\partial v_{3|i}}{\partial x^{j}}-v_{3|k}\gamma_{ji}^{k}+v_{k|i}{b^{k}}_{j}\right)M^{ij}-v_{k|i}{b^{k}}_{j}M^{ij}\\ & =-v_{3|i}S^{3i}+l_{3i|j}M^{ij}-v_{k|i}{b^{k}}_{j}M^{ij}. \end{array}$$

Using this we can express conservation of energy as

D 9 $$\begin{array}{rl} \rho_{0}\frac{\partial E}{\partial t} & =S^{ai}v_{a|i}-S^{3i}v_{3|i}+M^{ij}l_{3i|j}-v_{k|i}{b^{k}}_{j}M^{ij}\\ & =S^{ij}v_{i|j}-{b^{i}}_{k}M^{jk}v_{i|j}+M^{ij}l_{3i|j}\\ & =(S^{ij}-{b^{i}}_{k}N^{jk})v_{i|j}+N^{ij}l_{3i|j}. \end{array}$$

We recognize the term in the brackets as the modified Piola–Kirchhoff stress tensor $\tilde{S}$. Recalling that this is symmetric we can write conservation of energy as

D 10 $$\rho_{0}\frac{\partial E}{\partial t}={\tilde{S}}^{ij}n_{ij}+N^{ij}l_{3i|j}.$$

Using the kinematic results derived in §3 we can write this as

D 11 $$\rho_{0}\frac{\partial E}{\partial t}={\tilde{S}}^{ij}{\dot{E}}_{ij}+N^{ij}{\dot{H}}_{ij}.$$

The fact that $\dot{H}$ is symmetric means that only the symmetric part of **N** contributes to this expression, but as is discussed in §5, **N** is assumed to be symmetric. Hence, we can write conservation of energy in component free form as

D 12 $$\rho_{0}\frac{\partial E}{\partial t}=\mathrm{tr}(\tilde{S}\dot{E})+\mathrm{tr}(N\dot{H}).$$
